# The role of lncRNAs in the tumor microenvironment and immunotherapy of melanoma

**DOI:** 10.3389/fimmu.2022.1085766

**Published:** 2022-12-19

**Authors:** Wencheng Zhou, Xuewen Xu, Ying Cen, Junjie Chen

**Affiliations:** Department of Burn and Plastic Surgery, West China Hospital, Sichuan University, Chengdu, Sichuan, China

**Keywords:** lncRNAs, melanoma, immunotherapy, tumor microenvironment, targeted therapy

## Abstract

Melanoma is one of the most lethal tumors with highly aggressive and metastatic properties. Although immunotherapy and targeted therapy have certain therapeutic effects in melanoma, a significant proportion of patients still have drug resistance after treatment. Recent studies have shown that long noncoding RNAs (lncRNAs) are widely recognized as regulatory factors in cancer. They can regulate numerous cellular processes, including cell proliferation, metastasis, epithelial-mesenchymal transition (EMT) progression and the immune microenvironment. The role of lncRNAs in malignant tumors has received much attention, whereas the relationship between lncRNAs and melanoma requires further investigation. Our review summarizes tumor suppressive and oncogenic lncRNAs closely related to the occurrence and development of melanoma. We summarize the role of lncRNAs in the immune microenvironment, immunotherapy and targeted therapy to provide new targets and therapeutic methods for clinical treatment.

## 1 Introduction

The incidence and mortality of melanoma have gradually increased over the past few decades (1). Currently, multiple therapeutic strategies, including surgical resection, radiotherapy and chemotherapy, immunotherapy, and biological and targeted therapy, have significantly improved the therapeutic effect of melanoma and prolonged the survival time of patients (2). Given a strong metastatic tendency, these treatments have very limited therapeutic efficacy in patients with advanced melanoma. Moreover, the occurrence and progression of melanoma have complex relationships with targeted genes and signaling pathways that influence the proliferation, migration, invasion and metastasis of tumor cells (3). Therefore, it is important to understand the molecular mechanism of melanoma progression to complement effective therapeutic strategies.

LncRNAs play regulatory roles in a variety of tumors and are widely involved in multiple biological processes, such as proliferation, migration, invasion, EMT process, cell cycle, apoptosis and chemoresistance. Compared with benign nevi and melanocytes, lncRNA-ATB is upregulated in human skin melanoma tissues and cells. It regulates cell proliferation, metastasis, cell cycle arrest and apoptosis by regulating miR-590-5p and YAP1 (4). In addition, lncRNA-XIST promotes the proliferation and migration of melanoma cells by decreasing the expression of PI3KRI and AKT and increasing the expression of Bcl-2 and Bax, which are considered key regulators of oxaliplatin resistance in melanoma progression (5). Additionally, lncRNA-LINC00518 could significantly promote the invasion, migration, proliferation, clonogenicity and metastasis of malignant melanoma cells and induce radioresistance by regulating the miR-33a-3p/HIF-1α negative feedback pathway (6). Therefore, there is an urgent need to find new breakthroughs, such as specific lncRNAs, to improve melanoma therapeutic effects.

The communication between cancer cells and their surrounding microenvironment is very important in many tumors. LncRNA-NEAT1 promoted cell proliferation and migration by regulating the miR‐495‐3p/E2F3 axis and activated the EMT process and immune responses through the miR-200b-3p/SMAD2 pathway in melanoma (7, 8). Moreover, in melanoma cells with low FOXF1-AS1 expression, the expression of immune-related genes was downregulated, and the activity of inflammation and Wnt signaling pathways were also changed (9). Additionally, the expression of lncRNA-SNHG15 can be modulated by palbociclib and alleviate temozolomide resistance by regulating the CDK6/miR-627 pathway and reducing M2 polarization of glioma-associated microglia, providing evidence for treatment with temozolomide resistance with the use of CDK6 inhibitors (10).

Immune checkpoint inhibitors, especially anti-PD-1 (programmed death protein 1) antibodies, target the dysfunctional immune system and induce CD8-positive T cells to kill tumor cells, completely altering the treatment of various cancers, including advanced melanoma. In addition, targeted therapy for melanoma is primarily an appropriate treatment based on BRAF and NRAS mutational status. However, there are currently no highly sensitive and specific biomarkers to evaluate the therapeutic efficacy of immunotherapy and targeted therapy in patients with advanced melanoma. Therefore, lncRNAs may play an important role in melanoma immunotherapy and targeted therapy, serving as new therapeutic targets or drug sensitivity assessment markers. lncRNA-CRNDE (colon rectal neoplasia differentially expressed) promoted the cell invasion and apoptosis of melanoma by targeting CCL18, which was correlated with the expression of PD-L1 (programmed death ligand 1) and induced immunosuppression (11). Moreover, lncRNA-SNHG14 is upregulated in diffuse large B-cell lymphoma, and the SNHG14/miR-5590-3p/ZEB1 axis can also regulate the PD-1/PD-L1 checkpoint to promote the progression and immune evasion of tumor cells, which indicates that targeting SNHG14 may be a potential target to improve the immunotherapeutic effect in tumors (12). Thus, it is of great clinical importance to elucidate the therapeutic effect and molecular mechanism of lncRNAs in melanoma immunotherapy and targeted therapy.

In this review, we summarized the regulatory mechanisms by which lncRNAs exert oncogenic and tumor suppressive functions in tumor progression, particularly in melanoma. Furthermore, we screened lncRNAs involved in the regulation of the tumor immune microenvironment, provided relevant evidence for their efficacy in promoting immunotherapy and targeted therapy, and discussed their potential therapeutic prospects.

## 2 Oncogenesis of melanoma

Most of malignant tumors have complex etiologies, poor treatment effects and short survival times. Their overall incidence in the world is rising every year and seriously threatening human health. According to the statistics of cancer incidence and mortality rate of 38 cancer sites and 185 countries or regions in the world, it is estimated that there were 19.3 million new cancer cases and approximately 10 million cancer deaths around the world in 2020 (13). In recent years, almost 75% of patients with malignant melanoma have relapsed one year after treatment, and the 3-year overall survival rate of patients with advanced malignant melanoma is less than 30% (14). The incidence and mortality rates of melanoma were 324,635 and 57,043, respectively (15). Therefore, there is an urgent need to find an effective treatment for advanced melanoma.

The two of the most critical factors in reducing melanoma mortality are early detection and prompt treatment (16). Surgical resection is considered the primary treatment for early-stage melanoma, but it still has the possibility of metastasis and affects long-term survival outcomes (17). The US Food and Drug Administration (FDA)-approved treatments for metastatic melanoma, including immune checkpoint blocking antibodies (such as anti-CTLA-4 and anti-PD-1), have an effect on reducing population mortality (18). The anti-CTLA-4 drugs (including Ipilimumab) and anti-PD-1 drugs (including Nivolumab and Pembrolizumab) have therapeutic effects in advanced metastatic melanoma and are used as adjuvant therapy after surgery (19). In a study of 945 patients with stage III or IV melanoma, the overall survival after treatment with Nivoluma and Ipilimumad was 36.9 months and 19.9 months respectively, while the overall survival of Nivoluma and Ipilimumad combination was more than 60 months (20). The immune responses induced by anti-CTLA-4 and anti-PD-1 checkpoint blockade are driven by distinct cellular mechanisms. Anti-PD-1 mainly induces an increase in specific tumor-infiltrating exhausted-like CD8 T-cell populations, while anti-CTLA-4 predominantly induces the expansion of an ICOS^+^ Th1-like CD4 effector cell subset and binds to specific subsets of exhausted-like CD8 T cells (21). PD-1 inhibitors have become an adjuvant treatment for stage III or IV melanoma patients after surgical resection, and immune checkpoint therapy may become an extremely effective treatment in the future (22).

Transcriptome sequencing analysis of tissue samples from melanoma patients indicated that mutations closely related to melanoma progression mainly included BRAF mutation, NRAS mutation and NF1 mutation (23). These three mutations are found in most skin melanomas (about 94%) and can activate the downstream Ras/Raf/MEK/ERK axis (MAPK signal pathway) (24). Among them, BRAF mutation exists in more than 60% of skin melanomas and promotes the occurrence and development of tumors (25). ATF-3, a cyclic APM-dependent transcription factor, is significantly decreased in human metastatic melanoma cell lines. Overexpression of this gene downregulates the ERK and AKT signaling pathways, upregulates apoptosis-related genes, and reduces melanoma metastasis (26). In addition, silencing MED27 (as a potential melanoma target) leads to a decrease in iNOS expression by inhibiting the activity of a series of key proteins in the NF-κB signaling pathway and is accompanied by the inhibition of melanoma cell proliferation, induction of apoptosis and regulation of the cell cycle by changing the activity of the PI3K/AKT, MAPK/ERK and Bax/Cyto-C/Caspase-dependent apoptotic pathways (27). Moreover, some genes were found to improve the therapeutic effect of chemotherapy drugs on metastatic melanoma by regulating these target genes and signaling pathways. The expression of SEMA6A protein was higher in melanoma tissues from BRAF-mut patients than in melanoma tissues from BRAF-wt patients. In addition, SEMA6A regulates actin cytoskeleton remodeling through RhoA-dependent activation of YAP in BRAF-mut melanoma cells. Dabrafenib/trametinib treatment helps melanoma cells escape from the microenvironment, which may be a predictor of the effectiveness of dual BRAF/MEK (mitogen-activated protein kinase kinase) inhibitors in treating melanoma (28). Thus, the in-depth study of target genes and signaling pathways can improve the therapeutic effect in melanoma.

## 3 LncRNAs in carcinogenesis

**Figure 1:**
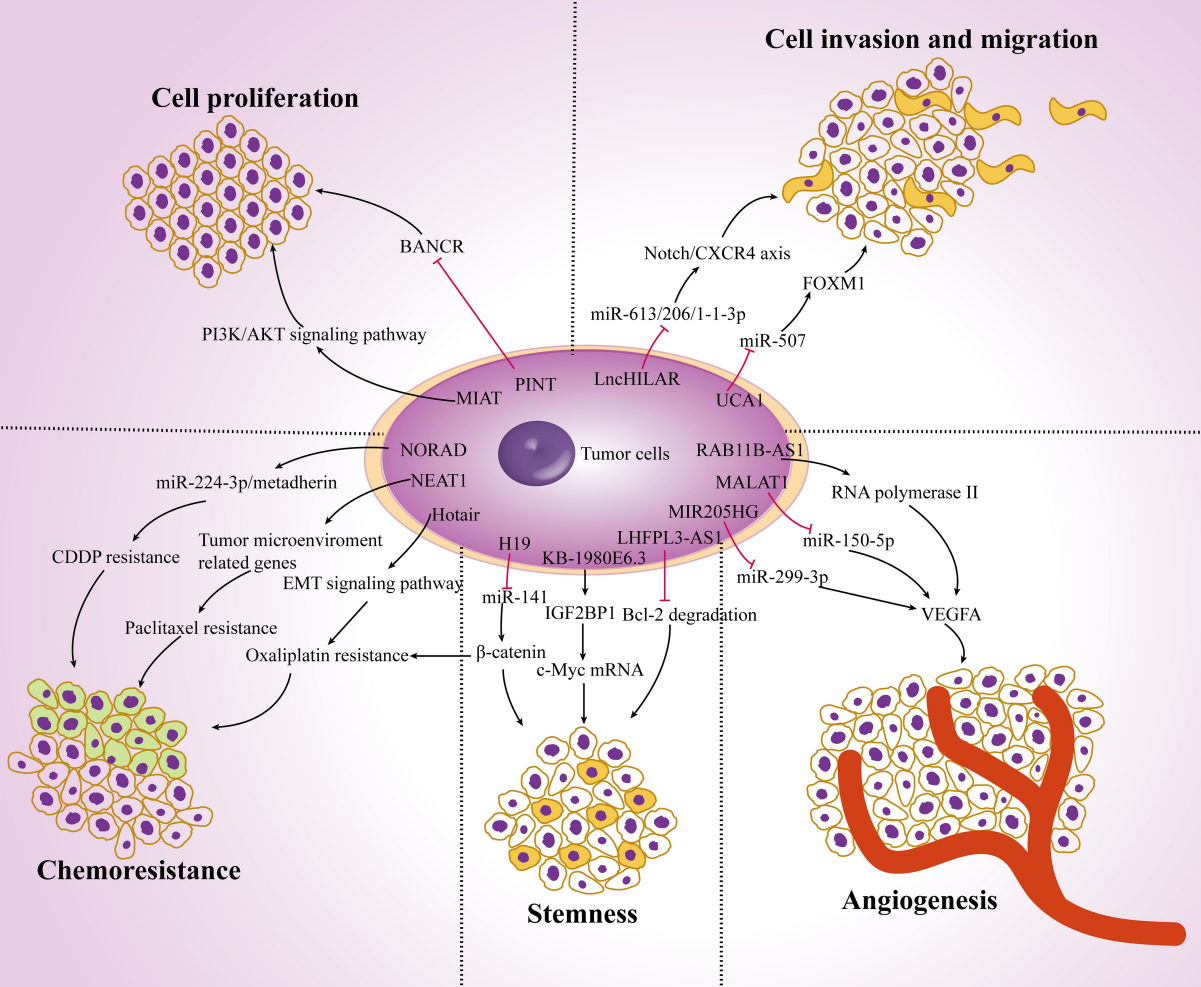
The role of lncRNAs in carcinogenesis. Dysregulation of lncRNAs in melanoma cells affects tumor cell proliferation, invasion and migration, angiogenesis, stemness and chemoresistance by targeting multiple genes and ultimately regulate tumor progression.

More than 90% of transcripts have not been translated into proteins in the human genome (known as noncoding RNA). Noncoding RNAs are a class of regulatory molecules that play a crucial role in regulating gene expression and are closely related to the progression of multiple diseases, especially different types of cancer. Both lncRNAs and miRNAs belong to the non-coding RNA. The difference is that the lncRNAs is longer than 200nt. They all regulate the expression of target genes. In particular, they are expected to be combined in the diagnosis and treatment of melanoma (29). Numerous studies have shown that lncRNAs play a key role in the initiation and development of cancer, participating in biological processes such as tumor cell proliferation, metastasis, EMT process, stemness, angiogenesis, chemotherapy resistance, and regulation of the tumor microenvironment (**Figure 1**).

LncRNAs can regulate a few target proteins related to tumor proliferation and metastasis. Hypoxia-induced lncRNA lncHILAR promotes cell invasion and migration by acting as a ceRNA for miR-613/206/1-1-3p, thus resulting in the upregulation of the Notch/CXCR4 axis (30). The expression of lncRNA-BASP1-AS1 was up-regulated in melanoma tissues. BASP1-AS1 interacts with YBX1 and recruit it into the promoter of Notch3 to activate the transcription of multiple oncogenes, including c-MYC, PCNA and CDK4, and promote the proliferation, migration and invasion of A375 and SK-MEL-2 cells (31). LncRNA-MALAT1 knockdown downregulates the expression of vascular endothelial growth factor A (VEGFA), enhances the expression of miR-150-5p and changes the proliferation and migration ability of vascular endothelial cells (32). Tumor stem cells also promote tumor proliferation and metastasis. For example, the expression of lncRNA-NR2F1-AS1 is increased in dormant mesenchymal-like stem cells. It mediates the translation of NR2F1 and inhibits the transcription of ΔNp63, thereby reducing the tumorigenicity and enhancing the dormancy of cancer cells (33). Moreover, overexpression of lncRNA-KB-1980E6.3 maintains the stemness of cells and enhances c-Myc mRNA stability by interacting with IGF2BP1, promoting tumorigenesis in a hypoxic microenvironment (34).

Accumulating studies have shown that lncRNAs play an important role in chemoresistance. lncRNA-MALAT1 increased drug resistance and promoted tumor cell proliferation by affecting the expression of cyclin D1, p-PI3K and p-Akt, as well as regulating the EMT process by targeting ZEB2, YAP, Vimentin and E-cadherin (35). In addition, lncRNA-H19 delivered by exosomes from carcinoma-associated fibroblasts (CAFs) can competitively bind miR-141 and activate the expression of β-catenin protein, thereby promoting the stemness and chemoresistance (36). LncRNAs change the therapeutic effects of multiple drugs on cancer. LncRNA-Hotair regulates the EMT-related signaling pathway by altering hypoxia-induced oxaliplatin resistance (37). Overexpression of lncRNA-NORAD affects the EMT process by interacting with hsa-miR-125a-3p, thus promoting the invasion and migration *in vitro* and *in vivo (* 38). Meanwhile, researchers also found that NORAD regulates the miR-224-3p/metadherin axis to increase the expression of β-catenin, thereby enhancing the CDDP resistance in tumor cells (39). In addition, lncRNA-NEAT1 directly targets the expression of many prometastatic genes and tumor microenvironment-related genes (such as STAT3, WNT7A and VEGF-A) by interacting with miR-361, while miR-361 can inhibit tumor proliferation, invasion, stemness and paclitaxel resistance (40). Moreover, lncRNA-SAMMSON is highly expressed in doxorubicin-resistant cancer cells, resulting in metabolic recombination, reduced production of mitochondrial ROS, increased mitochondrial replication, transcription, and translation, and reduced resistance to chemotherapy (41).

LncRNAs interact with the tumor microenvironment and affect cancer progression. LncRNA-H19 is considered to be a key lncRNA in CAFs. It is an important component of the tumor microenvironment. It can affect proliferation, migration and glycolysis by regulating miR-675-5p and PFKFB3 (42). Most importantly, lncRNAs can affect the function of a variety of immune cells. LncRNA-LINC00301 changes the amount of regulatory T cells and CD8+ T cells by regulating TGF-β, promotes cell proliferation, cell migration and invasion, releases cell cycle arrest, and reduces cell apoptosis in tumor cells (43). LncRNA-SATB2-AS1 is downregulated in tumor tissues and suppresses metastasis by regulating the expression of Th1-type chemokines and the number of immune cells (44). Recent studies have found that macrophages can also be regulated by lncRNAs in the tumor microenvironment. LncRNA-LNMAT1 induces the upregulation of CCL2 and recruits a large number of macrophages into tumor cells, which then promotes the secretion of VEGF-C and enhances tumor metastasis (45). LncRNA-HOMER3-AS1 modulates proliferation, migration, invasion and apoptosis in tumor cells and enhances M2 macrophage recruitment by activating the Wnt/β-Catenin signaling pathway and CSF-1 expression (46). Moreover, lncRNA-CRNDE can promote M2 macrophage polarization and indirectly modulate angiogenesis-related proteins such as VEGF, VEGFR2, Notch1 and Dll4, which is consistent with the regulatory mechanism in the tumor immune microenvironment (47). Taken together, these data indicate that lncRNAs are involved in carcinogenesis and may become a potential diagnostic target for malignant tumors.

## 4 LncRNAs as regulators in melanoma

### 4.1 Tumor suppressor lncRNAs in melanoma

Some lncRNAs have become tumor suppressors because they can affect the proliferation and metastasis of melanoma by competitively binding miRNAs and regulating downstream related signaling pathways, such as the Wnt and Hippo signaling pathways (**Table 1**). CASC2 is a lncRNA downregulated in a variety of cancer types, including endometrial cancer, lung cancer, gastric cancer and colorectal cancer, which exerts tumor suppressor effects through various mechanisms, such as inhibiting the Wnt/β-Catenin signaling pathway (79). Zhang Y et al. found that the overexpression of CASC2 in melanoma cells inhibited cell proliferation, migration and invasion by regulating miR-18a-5p and its target gene RUNX1 (48). LncRNA-linc00961 is also downregulated in cutaneous melanoma tissues compared to benign nevi. It restrains proliferation and promotes apoptosis in melanoma cells by regulating the miR‐367/PTEN axis (49). As reduced lncRNA-HOXA11-AS expression regulates the miR-152-3p/ITGA9 axis and inhibits the proliferation, metastasis, apoptosis and EMT of melanoma cells, it can be used as a biomarker for the diagnosis and treatment of cutaneous melanoma (50).

**Table 1:** Upregulated and Downregulated LncRNAs in Melanoma.

lncRNA Name	Expression		Functions	Targets and signaling pathways	Role	References
ATB	Increased		regulate proliferation, metastasis, cell cycle arrest and apoptosis	miR-590-5p; YAP1	Oncogenic LncRNAs	(4)
XIST	Increased		promote proliferation, migration and oxaliplatin resistance	PI3KRI; AKT; Bcl-2; Bax	Oncogenic LncRNAs	(5)
LINC00518	Increased		promote proliferation, invasion, migration, and induce radioresistance	miR-33a-3p; HIF-1α	Oncogenic LncRNAs	(6)
NEAT1	Increased		promote proliferation, migration, EMT process and immune responses	miR‐495‐3p; E2F3; miR-200b-3p; SMAD2	Oncogenic LncRNAs	(7, 8)
CRNDE	Increased		promote invasion and apoptosis	CCL18	Oncogenic LncRNAs	(11)
CASC2	Decreased		inhibit proliferation, migration and invasion	miR-18a-5p; RUNX1	Tumor suppressor lncRNA	(48)
Linc00961	Decreased		restrain proliferation and promote apoptosis	miR‐367; PTEN	Tumor suppressor lncRNA	(49)
HOXA11-AS	Decreased		inhibit proliferation, metastasis, apoptosis and EMT process	miR-152-3p; ITGA9	Tumor suppressor lncRNA	(50)
MEG3	Decreased		affect metastasis, apoptosis, cell cycle and enhance the chemosensitivity to cisplatin and 5-FU	miR-206; SOX4; miR-499-5; CYLD	Tumor suppressor lncRNA	(51, 52)
GAS5	Decreased		regulate G1/S cell cycle, apoptosis, reactive oxygen species and redox balance	gelatinases A and B	Tumor suppressor lncRNA	(53, 54)
NKILA	Decreased		block tumor growth and metastasis	NF-κB	Tumor suppressor lncRNA	(55)
CPS1-IT1	Decreased		control EMT and angiogenesis	BRG1; Cyr61	Tumor suppressor lncRNA	(56)
LINC-PINT	Decreased		inhibit proliferation and metastasis	BANCR; PCNA; CDK1; CCNA2; AURKA	Tumor suppressor lncRNA	(57, 58)
H19	Increased		affect proliferation, invasion, migration, apoptosis, G0 / G1 phase arrest and sensitivity to cisplatin	PI3K/AKT signaling pathway; NF-κB signaling pathway	Oncogenic LncRNAs	(59, 60)
MIAT	Increased		promote proliferation, migration and invasion	TCF12; NFAT5; PI3K/AKT signaling pathway	Oncogenic LncRNAs	(61, 62)
PVT1	Increased		promote proliferation and metastasis	EZH2; miR-200c	Oncogenic LncRNAs	(63, 64)
MALAT1	Increased		regulate proliferation, migration, invasion and cell apoptosis	miR-34a; c-Myc/Met; miR-23a	Oncogenic LncRNAs	(65, 66)
UCA1	Increased		regulate proliferation, invasion, migration, metastasis and cell cycle arrest	miR-507	Oncogenic LncRNAs	(67)
Gm31932	Increased		affect cell cycle arrest and melanoma differentiation	miR-344d-3-5p; Prc1; Nuf2	Oncogenic LncRNAs	(68)
SRA	Increased		promote EMT progression and distal metastasis	p38; CCL21; β-catenin; N-cadherin	Oncogenic LncRNAs	(69)
LHFPL3-AS1	Increased		encourage the stemness	Bcl-2	Oncogenic LncRNAs	(70)
MIR205HG	Increased		promote the angiogenesis	miR-299-3p; VEGFA	Oncogenic LncRNAs	(71)
PURPL	Increased		inhibit autophagy and reduce cell death	mTOR; ULK1; AMPK signaling pathway	Oncogenic LncRNAs	(72)
TUG1	Increased		promote the proliferation and metastasis, inhibit apoptosis and improve the chemosensitivity to cisplatin and 5-FU	miR-29c-3p; RGS1; Bcl-2; MMP-9; cyclin D1	Oncogenic LncRNAs	(73, 74)
orilncl	Increased		promote cell proliferation	MAPK signaling pathway	Oncogenic LncRNAs	(75)
SAMMSON	Increased		promote tumor growth and tolerant to vemurafenib	CARF; p53	Oncogenic LncRNAs	(76, 77)
MIRAT	Increased		affect drug resistance	IQGAP1; MAPK signaling pathway	Oncogenic LncRNAs	(78)

In addition to exerting tumor suppressor functions, lncRNAs also play a crucial role in influencing drug sensitivity *via* ceRNA regulation. LncRNA-MEG3 affects the differentiation of cancer stem cells and the metastasis of melanoma by inhibiting miR-206 and SOX4. MEG3 also regulates the expression of miR-499-5 and CYLD. Thus, it affects proliferation, invasion, migration, apoptosis and cell cycle processes and enhances the chemosensitivity of melanoma cells to cisplatin and 5-FU treatment (51, 52). When the expression of lncRNA-TINCR is decreased in metastatic melanoma, its downregulation promotes the expression level of proliferation-, migration- and invasion-related marker genes and increases its resistance to drugs such as BRAF and MEK inhibitors in melanoma progression (80). In addition, TINCR regulates the expression of LATS1 (a target of miR-424-5p) to activate the Hippo signaling pathway and to inhibit the activity of Yes-1-related transcriptional regulators, thus playing a tumor suppressor role in the development of cutaneous melanoma (81).

Tumor suppression-associated lncRNAs suppress melanoma progression through multiple mechanisms, such as the regulation of downstream target proteins and affecting other long noncoding RNAs. LncRNA-GAS5 plays an antitumor role by regulating gelatinases A and B in melanoma metastasis and promotes the proliferation of melanoma cells by regulating the G1/S cell cycle, apoptosis, reactive oxygen species and redox balance (53, 54). LncRNA-NKILA plays a role in preventing tumor growth and inhibiting metastasis in melanoma, breast cancer and other types of solid tumors, while the expression of NKILA is enhanced by the nuclear factor NF-κB (55). Recent studies have found that lncRNA-CPS1-IT1 is recognized as a tumor suppressor factor in several cancers, including melanoma. The competitive binding of CPS1-IT1 to BRG1 inhibits the expression of Cyr61 (an angiogenic factor involved in tumor metastasis) and works together to control the EMT and angiogenesis of melanoma cells (56). Moreover, the expression of lncRNA p53-induced transcript (LINC-PINT) was decreased in melanoma tissues compared to adjacent tissues, while LINC-PINT overexpression downregulated the expression of lncRNA-BANCR in melanoma cells to regulate cell proliferation (57). Additionally, LINC-PINT inhibits the growth and metastasis of melanoma by regulating the epigenetics of target genes, including PCNA, CDK1, CCNA2 and AURKA (58).

### 4.2 Oncogenic LncRNAs in melanoma

The other lncRNAs may serve as oncogenic lncRNAs because they regulate a variety of signaling pathways related to melanoma progression. LncRNA-H19 was upregulated in melanoma tissues compared to adjacent normal tissues. Furthermore, its expression in metastatic melanoma tissues was higher than that in orthotopic tumor tissues (59). Knockdown of H19 affects melanoma cell growth, invasion, migration, apoptosis, G0/G1 phase arrest and sensitivity to cisplatin (60). Functionally, downregulation of H19 mediates the inhibition of the PI3K/AKT signaling pathway and NF-κB signaling pathway, thereby inhibiting the progression of melanoma (82). LncRNA-BANCR (BRAF-activated long noncoding RNA) participates in the occurrence and development of melanoma by reducing the interaction with miR-204 and activating the Notch2 signaling pathway and promotes its expression in melanoma tissues and cell lines (83). The overexpression of lncRNA-MIAT obviously promotes the proliferation, migration and invasion of melanoma cells by regulating the PI3K/AKT signaling pathway and can also strengthen the interaction between TCF12 and the NFAT5 promoter region to promote the progression of melanoma (61, 62).

In oncogenic lncRNAs promoting melanoma development, microRNAs play a regulatory role. Accumulating studies have shown that lncRNA-PVT1 (named plasmacytoma variant translocation 1) is upregulated in melanoma tissues compared to adjacent normal tissues, and PVT1 levels are significantly higher in the serum of melanoma patients than in healthy individuals (63). In terms of molecular regulation, PVT1 promotes the occurrence and metastasis of melanoma by regulating the expression of EZH2 and miR−200c (64). The level of lncRNA-MALAT1 in melanoma was significantly higher than that in paired adjacent normal tissues, which affects the expression of c-Myc/Met by regulating a competing endogenous RNA of miR-34a and regulates cell proliferation, migration, invasion and cell apoptosis by miR-23a (65, 66). Additionally, the expression of lncRNA-UCA1 was upregulated in melanoma tissues compared to normal tissues, while the downregulation of UCA1 was controlled by direct binding with miR-507, resulting in cell proliferation, invasion, migration, metastasis and cell cycle arrest inhibition (67). Moreover, integrative transcriptome analysis demonstrated that lncRNA-Gm31932 has definite effects on cell cycle arrest and melanoma differentiation through the miR-344d-3-5p/Prc1 (and Nuf2) axis (68). LncRNA-HnRNPK (heterogeneous nuclear ribonucleoprotein K) acts as a ceRNA for miR-147a and regulates LINC00263, thus accelerating malignant capabilities by targeting CAPN2 (84).

LncRNAs affect the occurrence and development of melanoma by regulating a variety of biological processes, such as stemness, angiogenesis, autophagy and drug resistance. SRA, known as the steroid receptor RNA activator, is a lncRNA encoding the conserved protein SRAP. Its expression is upregulated in melanoma tissues compared to normal tissues. In melanoma cells, the deletion of SRA induces the activation of p38 and inhibits EMT process and distal metastasis by increasing the expression of CCL21 and reducing the expression of β-catenin and N-cadherin (69). Moreover, lncRNA-LHFPL3-AS1 was screened out by analyzing differentially expressed genes between stem cells and nonstem cells in melanoma, which encouraged the stemness of melanoma stem cells by inhibiting the degradation of Bcl-2 (70). The expression levels of lncRNA-MIR205HG were significantly upregulated in melanoma tissues and cells compared to normal skin tissues and cells. In addition, MIR205HG directly binds to miR-299-3p, and miR-299-3p then interacts with the 3’UTR of VEGFA mRNA to promote angiogenesis in melanoma (71). The direct interaction of lncRNA-PURPL (p53 upregulated regulator of p53 levels) with mTOR and ULK1 promotes the phosphorylation of ULK1 at Ser757 to inhibit autophagy and reduce cell death, while the inhibition of PURPL induces autophagy and inhibits melanoma progression by regulating the AMPK signaling pathway and leading to the phosphorylation of ULK1 at Ser555 and Ser317 (72). In addition, it was recently reported that the expression of lncRNA-TUG1 was negatively correlated with prognosis in patients with gastrointestinal tumors, urinary system tumors and gynecological tumors, independent of overall survival in patients with head and neck tumors or melanoma (85). However, knockdown of TUG1 inhibited the growth and metastasis of melanoma cells by regulating miR-29c-3p and its target gene RGS1, as well as inducing apoptosis (73). Moreover, inhibition of TUG1 expression can downregulate Bcl-2, MMP-9 and cyclin D1 protein, reduce the growth of tumors in melanoma and improve the chemosensitivity of A375 cells to cisplatin and 5-FU (74).

## 5 LncRNAs in the immune microenvironment

**Figure 2:**
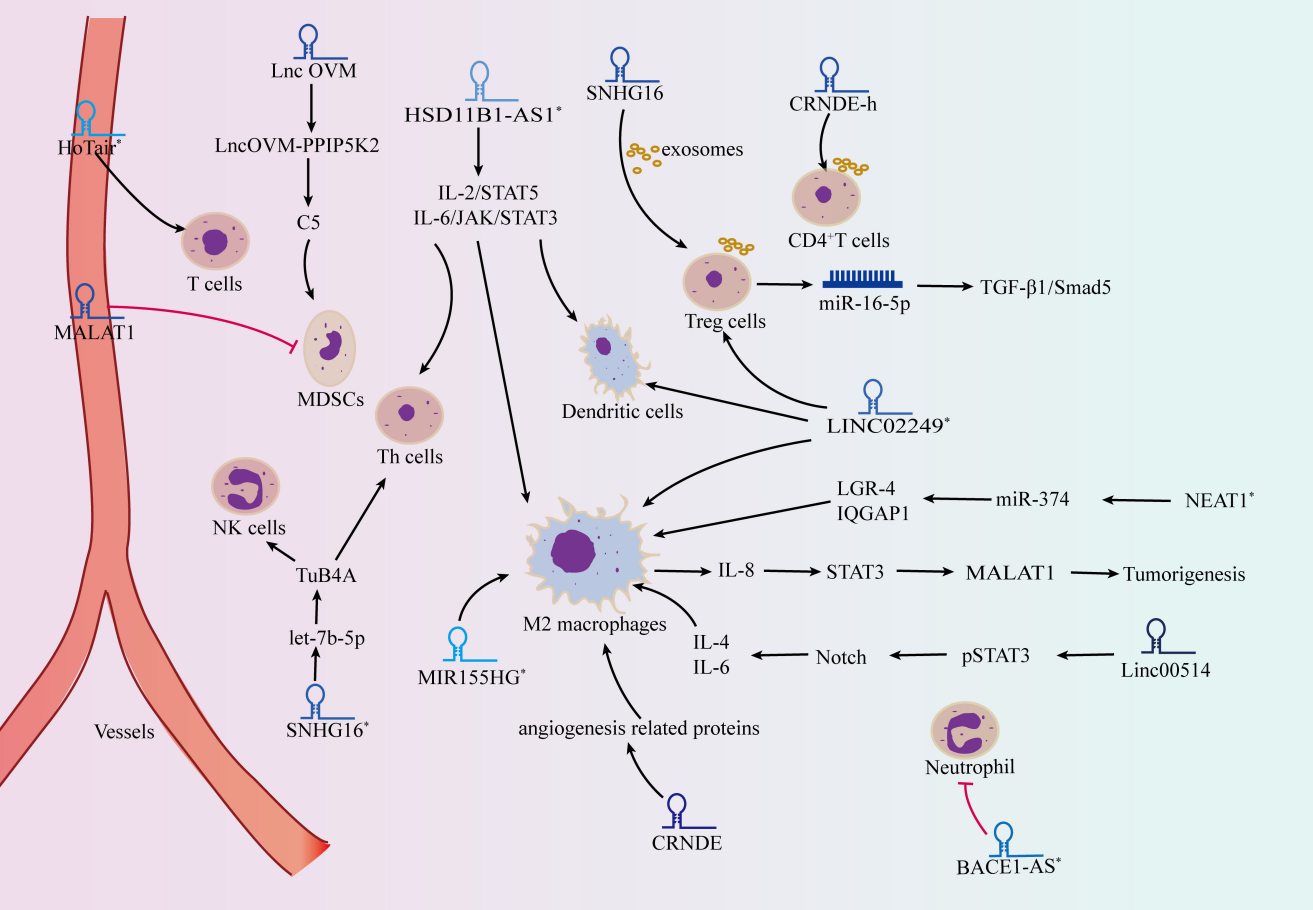
lncRNAs act as modulators in the tumor microenvironment. lncRNAs affect the occurrence and development of tumors through regulating the activity of immune cells, including CD8^+^ T cells, regulatory T cells (Tregs), T helper cells (Th cells), macrophages, myeloid-derived suppressor cells (MDSCs) and neutrophils. * refers to melanoma associated lncRNAs.

Immune-related lncRNAs can predict the prognosis of multiple tumors. They have the potential to become therapeutic targets for multiple tumors, including melanoma. Recently, researchers have analyzed the expression data of melanoma in the TCGA database and established a prediction model between immune-related lncRNAs and the survival status of melanoma (86, 87). The analysis in TCGA database indicated that 6 differentially expressed m7G-related lncRNAs have been identified, and a prognostic model was constructed for predicting the tumor growth, metastasis and survival status of patients (88). Another study found that a number of glycolysis-correlated lncRNAs show pivotal clinical effects by oncogenic pathways such as EMT and immune-related regulation (89). Based on next-generation sequencing technology, Yang et al. constructed a new immune-related lncRNA model and clarified that the high-risk group with low survival and low PD-L1 expression was associated with plasma B cell, monocyte, M2 macrophage, and neutrophil levels (90). The testis-specific lncRNA-RFPL3S was significantly downregulated in testicular germ cell tumors and correlated with the infiltration of immune cells such as T cells, B cells, NK cells and Th cells to predict the effect of immunotherapy (91). LncRNA-HSD11B1-AS1 was highly expressed in melanoma cells and promoted tumor proliferation, migration and invasion by targeting IL-2/STAT-5 and IL-6/JAK/STAT-3 signaling pathways. At the same time, immune invasion analysis showed that HSD11B1-AS1 affected the activation of T cells, Th cells, dendritic cells and B cells (92). And the overexpression of lncRNA-LINC02249 is associated with a shorter survival time in melanoma patients, and affects the immune infiltration of dendritic cells, Treg cells and macrophages (93). LncRNA-SNHG16 regulates mitochondrial function, cell metabolism and the immune infiltration of Th cells and NK cells by competitively binding with let-7b-5p and targeting TUB4A (94). Moreover, lncRNAs in immune cells also play important roles in the occurrence and development of cancer. Transcriptome sequencing analysis of lncRNAs in immune cells showed that the lncRNA expression profiles of T cells and monocytes differed between normal human and melanoma patients. These results provide new possibilities for the regulatory mechanisms of different immune cells, helping to accelerate the immunotherapy of specific cell types in melanoma (95) (**Figure 2**).

A multiomic integrative assessment including lncRNAs was performed to identify key molecular characteristics for the transcriptomic status of melanoma cells, which was significantly correlated with the therapeutic efficacy of checkpoint inhibitors and adoptive T cells (96). The detection of lncRNA-HOTAIR in the serum and intratumoral lymphocytes of metastatic patients suggested that it was involved in the regulation of the tumor microenvironment and could be used for the treatment of malignant melanoma (97). LncRNA-SNHG16 isolated from exosomes of tumor cells can increase the expression of CD73 in γδ1 Treg cells by regulating the TGF-β1/Smad5 signaling pathway (98). LncRNA-CRNDE-h is also abundant in tumor exosomes and participates in tumor progression by mediating ubiquitination and degradation of RORγt, promoting Th17-cell differentiation and affecting the activity of the IL-17 promoter (99). Single-cell sequencing analysis has identified noncoding IL-4 RNA (IL4nc), which can promote the production of IL-4 protein in Th2 cells by posttranscriptional regulation (100).

M2 macrophages can stimulate IL-8 secretion and promote the STAT3 signaling pathway in tumor progression. Subsequently, STAT3 binds to the lncRNA-MALAT1 promoter region and transcriptionally activates MALAT1 expression, inhibiting cell proliferation, invasion and tumorigenesis (101). Overexpression of lncRNA-linc00514 promotes the phosphorylation of the transcription factor STAT3, activates the Notch signaling pathway, facilitates the secretion of IL-4 and IL-6, and finally induces M2 polarization of macrophages (102). Furthermore, lncRNAs, including SNHG12, PACERR and HITT, function as key regulators of tumor-associated macrophages, regulating tumor cell proliferation, invasion and migration by altering the number of M2-polarized cells and contributing to immune escape (103–105). LncRNA-MIR155HG regulates the infiltration of macrophages and the balance of M1/M2 macrophages in tumor microenvironment to affect cell cycle and apoptosis as well as promoting melanoma progression (106). And lncRNA-NEAT1 derived from exosomes can inhibit miR-374, promote the expression of LGR4 and induce the recruitment of M2 macrophages to accelerate melanoma (107).

Neutrophils are also involved in the immunomodulatory process of lncRNA in tumors. While lncRNA-BACE1-AS is an immune-related factor in the tumorigenesis of melamona, its expression levels are negatively correlated with the neutrophil content (108). Neutrophil extracellular traps (NETs) generated in the tumor microenvironment promote the EMT process and metastasis by promoting the expression of lncRNA-MIR503HG and activating the downstream NF-κB/NLRP3 signaling pathway (109). Therefore, lncRNAs play an important role in the regulation of the immune microenvironment, thus affecting tumor progression.

Myeloid-derived suppressor cells (MDSCs) are precursors of dendritic cells, macrophages and granulocytes, which can inhibit the immune response of tumors and facilitate the formation of the tumor microenvironment. LncRNA-MALAT1 resulted in a significant reduction in MDSC numbers and decreased peripheral blood mononuclear cells in patients with malignant tumors (110). LncRNA-LncOVM maintains the stability of PPIP5K2 by inhibiting ubiquitination degradation and promoting the secretion of complement C5, thus allowing complement C5 to attract MDSC infiltration in the tumor microenvironment and promote tumor metastasis (111). Recent studies have found that lncRNAs also play a role in regulating the development and function of polymorphonuclear bone marrow-derived suppressor cells (PMN-MDSCs). For example, lncRNA-AK036396 is highly expressed in PMN-MDSCs, and its downregulation can weaken the stability of Fcnb protein through the ubiquitin−proteasome pathway, thereby affecting the maturation and immunosuppressive function of PMN-MDSCs in tumors (112). Although the interaction between lncRNAs and immune cells (include T cells, macrophages, neutrophils) has been reported in several studies, the interaction between lncRNAs and MDSCs is still unclear. The function and mechamism of lncRNAs in regulating the immune microenvironment in melanoma still needs further research.

## 6 LncRNAs as potential therapeutic targets

### 6.1 Immunotherapy

The use of immune checkpoint inhibitors to enhance the T-cell immune response holds great promise in tumor immunotherapy. However, the effect of immune checkpoint inhibition in patients with solid tumors is very limited, and the mechanism and efficacy of this treatment of solid tumors remain unclear. Computational analysis indicated that lncRNAs play an important role in evaluating the tumor immunotherapy response, and their binding to specific immune checkpoint factors can serve as biomarkers of the immune checkpoint inhibitor response (113). Therefore, it is necessary to elucidate the mechanism of action of lncRNAs and explore new combined strategies for immunotherapy.

LncRNAs transcribed from PD-L1 gene sites also affect the effectiveness of tumor immunotherapy. PD-L1-lnc, a long noncoding RNA subtype produced by alternative splicing of PD-L1 mRNA, can promote the progression of tumor cells by enhancing the transcriptional activity of c-Myc in human lung adenocarcinoma. Its depletion coupled to PD-L1 blockade may be used for tumor suppression (114). LncRNA-INCR1 (interferon-stimulated noncoding RNA 1) also transcribed from the PD-L1 locus promotes the expression of PD-L1, JAK2, and several IFNγ-related genes, which can regulate the sensitivity of tumor cells to cytotoxic T-cell-mediated killing and affect the therapeutic effect of CAR T-cell therapy (115).

As a monoclonal therapy, PD-1 has been used in the treatment of multiple tumors, including melanoma, and can predict the survival of patients. Recent studies have described the characteristics of tumor infiltrating immune-related lncRNAs (Ti-lncRNAs) and have found a better efficacy of anti-PD-1 treatment in melanoma patients with a low Ti-lncRNA score (116). WGCNA indicated that 15 lncRNAs, such as NARF-AS1 and LINC01126, were identified to predict the prognosis of melanoma patients treated with anti-PD-1 (117). By regulating the expression of miR-33a-5p and miR-330b-5p, lncRNA-LINC01140 promotes c-Myc expression, suppresses cisplatin-induced apoptosis and promotes cell proliferation and metastasis. In addition, this lncRNA directly decreased the expression of miR-377-3p and miR-155-5p, resulting in increased PD-L1 expression. Knockdown of lncRNA-LINC01140 in combination with CIK treatment can inhibit the expression of PD-L1 in severe combined immunodeficiency mice and has the potential to become a more effective target for tumor growth inhibition (118). Additionally, Q Hu et al. found that the level of lncRNA-LINK-A was elevated and that the antigen peptide-loading complex was downregulated in triple-negative breast cancer patients with PD-1 blockade tolerance, which may provide a basis for the development of new combined immunotherapies and effective early prevention strategies (119). In addition, lncRNA-SNHG29 inhibits PD-L1 expression under treatment with simvastatin (considered a novel inhibitor of PD-L1) by mediating YAP activation and promoting the antitumor immune process, which clarifies the therapeutic implications of SNHG29 in an antitumor immune response (120). In the cytoplasm, lncRNA-IFITM4P directly binds to SASH1 and phosphorylates TAK1 (Thr187) to increase the phosphorylation of NF-κB (ser536), thus inducing the expression of PD-L1, inhibiting the activation of the immune system and increasing the immune escape of tumor cells. IFITM4P enhances the interaction of KDM5A with the PTEN promoter, resulting in reduced transcription of PTEN and upregulated PD-L1 expression, thus activating the therapeutic sensitivity of PD-1 in the nucleus of tumor cells (121). LncRNA-NORAD reduces the expression of miR-199a-5p in exosomes by inhibiting the expression of pri-miR-199a1, and pri-miR 199a1 suppresses the ATR/Chk1 pathway by targeting EEPD1, enabling cells to better respond to radiotherapy. At the same time, inhibiting the expression of NORAD can reduce the ubiquitination of PD-L1, thereby increasing sensitivity to radiation and anti-PD-1 therapy in a mouse model (122). Thus, lncRNAs are involved in PD-1/PD-L1-related immunotherapy and may become a target of combined therapy.

The method of editing lncRNAs in T cells has the potential to become a new antitumor immunotherapy. Recent studies have found that knockdown of lncRNA-NKILA in cytotoxic T lymphocytes regulates the sensitivity of T cells to activation-induced cell death by reducing the expression of NF-κB, thereby effectively inhibiting the growth of patient-derived xenografts from breast cancer in mice (123). Melanoma-overexpressed antigen 1 (MELOE-1), which is encoded by a long noncoding RNA in tumor cells and can specifically improve the tumor antigen of MELOE-1 through thapsigargin drug stimulation, enhances the ability of T cells to recognize melanoma cells (124). Additionally, exosome-related therapy can inhibit tumor progression by regulating the immune system of the organism. M1 macrophage-derived exosomal lncRNA-HOTTIP and M2 macrophage-derived exosomal lncRNA-AFAP1-AS1 affect tumor metastasis by modulating the miR-26a/ATF2 axis and miR-19a/b-3p/TLR5/NF-κB signaling pathway, respectively, which provides a potential strategy for tumor immunotherapy (125, 126). In summary, targeting tumors with immune checkpoint inhibitors (especially anti- PD-1/PD-L1) combined with lncRNA modulation is a promising method for tumor therapy.

### 6.2 Targeted therapy

**Figure 3:**
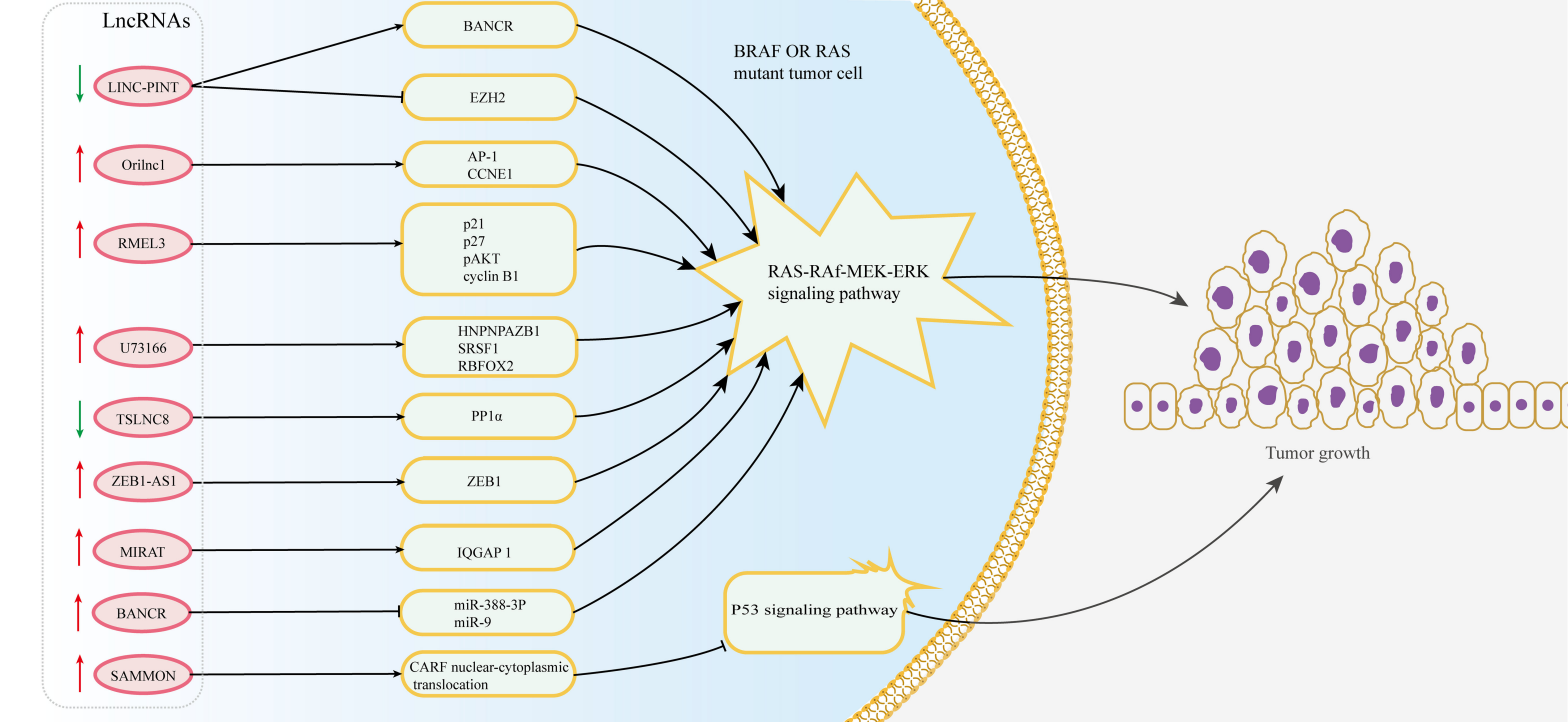
Multiple lncRNAs regulate resistance to BRAF inhibitors in melanoma. The dysregulation of lncRNAs targets the MAPK and p53 signaling pathway by regulating downstream target proteins in BRAF or RAS mutant melanoma cells, thus affecting the resistance of melanoma to BRAF inhibitors.

Approximately 66% of malignant melanomas have BRAF (B-RAF proto-oncogene) mutations, which lead to an increase in constitutive BRAF kinase activity and the MEK-ERK1/2 pathway and are necessary for proliferation, invasion and survival in melanoma cells (127). Recent studies have found that lncRNAs are associated with BRAF mutation and the growth of melanoma cells (**Figure 3**). LncRNA-ZEB1-AS1 is upregulated in melanoma cells and is related to the mutations of BRAF and RAS family genes, which can affect the invasion and migration of melanoma by activating the expression of ZEB1 (128). Moreover, a p53-induced long intergenic noncoding RNA (named LINC-PINT) affects the proliferation, migration and invasion of melanoma by interacting with the BRAF-activated noncoding RNA/MAPK pathway (129). Additionally, lncRNA-orilncl (the genetic target of RAS) was upregulated in BRAF mutant melanoma and promoted tumor cell proliferation and growth by regulating the RAS-RAF-MEK-ERK signaling pathway (75).

Although BRAF inhibitors have made great progress in the treatment of melanoma, the generation of drug resistance in tumor cells limits their efficacy. The experiment identified 11 lncRNA loci that induce resistance to BRAF inhibitors through genome-scale CRISPR activation screening and characterization (130). The expression of lncRNA-RMEL3 is significantly increased in BRAF V600E mutant melanoma cells and can be regulated by BRAF and MEK inhibitors. And its expression can promote colony formation in melanoma cells and the growth of subcutaneous xenografts in mice by inducing protein levels of p21, p27, pAKT and cyclin B1 (131). LncRNA-SAMMSON is a target of the transcription factor Sox10 and can interact with p32 to strengthen its role in targeting mitochondria and promoting cancer progression (132). The overexpression of SAMMSON made melanoma cells tolerant to the cytotoxicity induced by vemurafenib (functioning as an inhibitor of mutant BRAF kinase) by modulating the CARF/p53 axis (76, 77). Additionally, the intergenic lncRNA-U73166 changes the proliferation, migration and invasion abilities of melanoma cells and is also related to vemurafenib chemotherapy resistance (133). In addition, the expression level of lncRNA-TSLNC8 is downregulated in BRAF inhibitor-resistant melanoma cells, and its low expression attenuates the toxicity response of tumor cells to PLX4720 (a type of BRAF inhibitor). Mechanistically, TSLNC8 activates the MAPK signaling pathway by regulating the accumulation of PP1α in the cytoplasm and promotes the sensitivity of tumor cells to PLX4720, which enables melanoma patients to benefit from the combined treatment of PLX4720 and TSLNC8 (134). MIRAT, a novel cytoplasmic intergenic lncRNA, is upregulated in NRAS mutant melanoma and regulates the MEK scaffold protein IQGAP1 and MAPK signaling pathways to influence the drug resistance of tumor cells (78). Thus, it has the prospect to better explore the mechanism of drug resistance and improve the response to BRAF inhibitors.

Regulating the expression of lncRNAs by common drugs or oligonucleotides may be a potential way to inhibit the occurrence and development of melanoma. Researchers found that lncRNA-SLNCR interacts with AR and regulates the combination of AR- and EGR1-specific genomic sites, which cooperate with growth-related downstream regulatory genes to promote the proliferation of melanoma (135). Taking advantage of oligonucleotides binding to the AR N-terminal domain or AR RNA motif to block the interaction between SLNCR and AR represents a feasible therapeutic strategy in the process of melanoma (136). In addition, lncRNA-ZCCHC4 inhibits DNA damage-induced apoptosis by interacting with lncRNA-AL133467.2, and knockdown of this gene can enhance chemosensitivity to DDA in hepatocellular carcinoma cells, which is a potential target to improve the chemotherapeutic effect (137). Additionally, the lncRNA-POU3F3 expression level was elevated in dacarbazine-resistant melanoma cells, and knockdown of POU3F3 restored the sensitivity of cells to dacarbazine by secreting miR-650 and upregulating the expression of MGMT protein (138). Another novel therapeutic strategy, reprogramming abnormal lncRNA-ANRIL in gene clusters at chromosome 9p21, can significantly reduce the ability of tumor growth and metastasis (139). Therefore, targeting lncRNAs is a feasible way to inhibit the progression of melanoma.

## 7 Conclusion

Melanoma is one of the most rapidly progressing tumors with strong metastatic potential. Although a large number of genes involved in the tumor process have been found in melanoma, the specific molecular targets for their occurrence and development still need further research. Accumulating studies have found that lncRNAs play a key role in biological processes, including tumor proliferation, migration, invasion, cell cycle, apoptosis, stemness, EMT and chemoresistance. Based on the role of lncRNAs in melanoma, it can be used as a biomarker or a therapeutic target for the early diagnosis, prognosis and treatment of melanoma patients. Because lncRNAs can be secreted into the body fluids, the early diagnosis and speculated prognosis of melanoma can be performed painless by the lncRNAs analysis in the body fluids, compared with the invasive biohistopathological biopsies. For treatment, if the tumor suppressor lncRNA is downregulated, we might return it to normal function or even overexpression. Conversely, if the oncogenic lncRNA is upregulated in melanoma, we may suppress the oncogenic lncRNA. ASOs(antisense oligonucleotides), RNAi(RNA interference) and CRISPR(clustered regularly interspaced short palindromic repeats) are the main methods of downregulating lncRNAs. However, because the molecular mechanism of many lncRNAs is unclear and the interaction with functional partners, including proteins, is still uncertain, the development of lncRNA therapy is limited to a certain extent and needs further research.

Immunotherapy (such as immune checkpoint inhibitors) and targeted therapies (such as BRAF inhibitors) can exert certain therapeutic effects, but their effectiveness is usually limited by drug resistance. Reassuringly, a few lncRNAs can influence the therapeutic effect of immune checkpoint inhibitors in melanoma, and it is a feasible method to target tumors in combination with immune checkpoint inhibitors and lncRNA regulators. Furthermore, lncRNAs also play a regulatory role in alleviating the drug resistance caused by the use of BRAF inhibitors. Moreover, it is effective to eliminate the point mutation of the binding site between lncRNA and protein or use oligonucleotides to block the invasion of melanoma, which indicates that targeting lncRNA and its protein complexes has therapeutic prospects in melanoma. However, it is still unclear whether other novel lncRNAs are involved in immunotherapy and targeted therapy and whether they can be applied to clinical treatment. Therefore, elucidating the mechanism of lncRNAs in immunotherapy and targeted therapy and applying it to improve the effectiveness of drug therapy remain to be studied.

## Author contributions

Literature search and manuscript preparation: WZ. Concepts and paper design: XX, YC and JC. Manuscript review: JC. Authors contributed to the article and approved the submitted version.
